# Effect of first-line topical medications for the treatment of glaucoma on the ocular surface in a Latin American population

**DOI:** 10.1007/s10792-026-04205-5

**Published:** 2026-08-21

**Authors:** Oscar Luis Teherán Forero, Liliana María Hoyos Córdoba, Cristina Isabel Balmaceda Herrera, Stella Ortega Buelvas, Enrique Ramos Clason

**Affiliations:** 1 School of Medicine, Universidad del Sinú EBZ, Cartagena Sectional, Cartagena, Colombia; 2 Universidad del Sinú EBZ, Cartagena Sectional, Cartagena, Colombia; 3 Faculty of Nursing, Health Services Administration Program, University of Cartagena, Cartagena, Colombia

**Keywords:** Primary open-angle glaucoma, Ocular hypertension, Corneal thickness, Epithelial thickness, Ocular surface

## Abstract

**Background:**

First-line drug treatment is the cornerstone of glaucoma management. However, their adverse effects on the ocular surface have led to multiple retrospective studies in previously treated patients. This study evaluated these effects in patients who had never been exposed to these drugs.

**Purpose:**

To evaluate the effect of first-line drugs for the management of primary open-angle glaucoma and ocular hypertension on the cornea over one year of follow-up.

**Methods:**

An observational, longitudinal, and prospective study of patients from the Colombian Caribbean coast with no prior treatment or ocular alterations. The patients were evaluated with the OCT-Cirrus 6000, the Ocular Surface Analyzer (OSA), and the OSDI survey at one-year follow-up. The statistical analysis was performed using the Epi Info v.7.2 software.

**Results:**

A total of 88 eyes of 44 patients with an average age of 62.9 years were analyzed. Total corneal thickness (TCT) decreased from 537.6 to 533.8 µm, with no statistical significance, and total epithelial thickness (TET) decreased from 45.6 to 43.9 µm, a statistically significant difference. The OSDI survey increased from 7.3 to 13.4. The OSA showed a reduction in BUT from 8.3 to 6.9 s, the lipid layer from 3.8 to 3.0 nm, the tear meniscus height from 0.37 to 0.29 mm, and meibomian gland loss increased 12.6% at 1-year follow-up; all of these parameters were statistically significant.

**Conclusions:**

The findings reveal that antiglaucoma medications induce alterations in the ocular surface and cornea, and suggest that the greater the involvement of the corneal epithelium, the greater the symptoms reported by the patient.

## Introduction

Glaucoma is the leading cause of irreversible blindness worldwide and it is the second cause of blindness, after cataracts, according to the World Health Organization [1]. There are several risk factors associated with the development of this disease; however, to this date, the only modifiable factor identified is increased intraocular pressure (IOP). Currently, management guidelines for primary open-angle glaucoma (POAG) are aimed at reducing IOP. First-line options include antiglaucoma medications and selective laser trabeculoplasty [2, 3]. The use of antiglaucoma medications have been the cornerstone of POAG treatment for years. However, it is widely known that prolonged use can lead to adverse effects on the ocular surface [4, 5].

Dry eye is a multifactorial ocular surface disorder characterized by loss of tear film homeostasis and accompanied by ocular symptoms. Its primary etiology includes tear instability and damage to the ocular surface, factors that can be aggravated by exposure to external agents, such as antiglaucoma medications [6].

Among the most commonly used first-line medications are brimonidine, latanoprost, and timolol, which have been identified to this date, as the medications that have the most repercussions on the ocular surface [4, 7]. The chronic use of these medications has been associated to allergic reactions and the exacerbation of symptoms associated to dry eye, such as: discomfort, hyperemia and ocular itching [8, 9]. These discomforts often contribute to the abandonment of drug therapy, which makes it difficult to adequately control intraocular pressure, favoring the progression of glaucomatous disease, with the consequent risk of irreversible vision loss.

This situation has led several researchers to study the relationship between antiglaucoma medications and their effects on the ocular surface. However, in the majority of cases, these evaluations have been subjective, based mainly on symptoms refered by patients [7, 10]. Some recent studies have sought to establish this relationship in a more objective and quantifiable way, through the use of diagnostic tests that allow the affected areas to be identified more precisely [4, 5]. To this date, no prospective study has been reported that specifically evaluates the behavior over time of the layers of the cornea, mainly the corneal epithelium when exposed to antiglaucoma medication for the first time [4, 5, 10, 11].

With the advancements of the Cirrus® 6000 system, which integrates Swept Source technology, it allows for a more precise evaluation of epithelial and total corneal thickness. This device is capable of generating corneal thickness maps covering a diameter up to 9 mm, which significantly expands its diagnostic capacity by improving the penetrance and visualization of this structure [12]. Although previous studies have identified a decrease in the corneal epithelium associated with the use of antiglaucoma medication, the evolution of this alteration over time has not yet been consistently documented, specially in patients who start ocular hypotensive treatment for the first time, which is usually maintained in a chronic manner [4, 5, 10, 11].

In view of what has been previously stated and even though there are previous studies that have evaluated in isolation the effects of antiglaucoma medications on the ocular surface, the purpose of this study was to analyze fully and in an articulated manner, the behavior of the thickness of the corneal epithelium through the use of the OCT Cirrus 6000, the alterations on the ocular surface evaluated with the Ocular Surface Analyzer (OSA) and the description of the subjective symptoms reported by patients diagnosed with glaucoma through the Ocular Surface Disease Index (OSDI) questionnaire.

## Methods

This prospective, longitudinal, analytical, observational study was conducted at the Ophthalmology Clinic of Cartagena, Colombia. Patients were recruited from April 2023 to 2024 and they were all followed up for 12 months. It was approved by the Universidad del Sinu Medical Ethics Committee under the code OFT2023II-3C. The study met the ethical principles established in the Declaration of Helsinki, and informed consent was obtained from all participants.

*Sample* The population was made up of patients diagnosed with ocular hypertension or POAG in any of its stages.

The selection criteria were

1. Inclusion criteria
  Age over 18 years
  Patients diagnosed with OHT or POAG according to the 2021 European consensus [3] who were diagnosed for the first time and who had never used ocular hypotensive medications.
  Patients who met criteria for use of any of the following medications: latanoprost, timolol, and brimonidine.
  Absence of any previous topical ocular treatment in the last year.
  Absence of dry eye disease diagnosis in the last 5 years.
2. Exclusion criteria
  Any type of secondary glaucoma
  Change in the prescribed antiglaucoma medication.
  Presence of scarring or eye surgeries of any kind.
  Any type of connective tissue disease that requires treatment for the ocular surface.
  Use of eye drops other than those established in the treatment, including ocular lubricants.
  Patients who did not attend scheduled visits.
  Pregnancy

*Method* Patients who met the selection criteria were selected from the glaucoma service. This evaluation was performed by a single glaucoma specialist (OTF).

The diagnostic criteria for patients was established under the guidelines of the European Glaucoma Society of 2020 [2], in the following way:

1. *OHT* Patients with open angle by gonioscopy with IOP greater than 21 mmHg in 2 different measurements within one day, without structural or functional alteration of the optic nerve [2].
2. *POAG* Patients with progressive optic neuropathy with clinical characteristics of glaucomatous damage, evidenced by optic nerve tomography and with characteristic defects reported in campimetry. Presence of open angle under gonioscopy and absence of secondary etiology [2].

After diagnostic confirmation and before starting pharmacological treatment, all patients underwent an initial assessment which included measurement of epithelial and corneal thickness using the Cirrus® 6000 equipment (Dublin, CA, USA: Carl Zeiss Meditec, Inc.; 2020), followed by OSA analysis (SBM Sistemi, Turin, Italy; 2018) and finally, the OSDI survey was performed. These studies were repeated at 3, 6 and 12 months after the start of medical treatment. In each visit compliance with medical treatment was verified.

The results from the pachymetric and topographic analysis reported by the OCT Cirrus 6000 were taken into consideration as follows:

- *Ring 1* Corresponds to the central 2 mm area of the cornea.
- *Ring 2* Corresponds to the area between 2 and 5 mm peripheral to the cornea.
- *Ring 3* Corresponds to the area between 5 and 7 mm peripheral to the cornea.
- *Ring 4* Corresponds to the peripheral area between 7 and 9 mm from the cornea.
- *Total corneal thickness (TCT)* Corresponds to the average of the 3 concentric corneal rings.
- *Total epithelial thickness (TET)* Corresponds to the average of the 4 concentric epithelial rings.

In all cases, the therapeutic decision was made exclusively by the glaucoma specialist, who considered the specific contraindications of the medications, as well as the comorbidities and clinical characteristics of each patient. All antiglaucoma medications used in this study included preservatives such as benzalkonium chloride (BAK). The following were selected: Latanoprost (GAAP®, Laboratorios Sophia S.A. de C.V., Guadalajara, Mexico), Brimonidine (Brimoftal TQ®, Laboratorios TQ S.A., Bogotá, Colombia), and Timolol (Timolol TQ®, Laboratorios TQ S.A., Bogotá, Colombia).

The parameters evaluated with the OSA equipment were the following:

- *BUT* Reported in seconds (sec)
- *Lipid layer* Reported in nanometers of wavelength (nm)
- *Tear meniscus height* Reported in millimeters (mm)
- *Meibomian gland loss* Reported as a percentage (%).

All OSA examinations were performed by the same anterior segment specialist under standardized environmental conditions throughout the study to minimize measurement variability.

*Data analysis* The data obtained from the study were processed and analyzed using Epi Info software (version 7.2.6.0) and SPSS Statistics (version 25.0). Descriptive analysis of qualitative variables was performed by calculating absolute and relative frequencies. Quantitative variables were summarized using measures of central tendency (means) and dispersion (standard deviations). In addition, the mean reduction of each evaluated parameter was calculated.

Comparisons of the evaluated parameters at 90, 180, and 365 days of follow-up were performed using repeated-measures analysis of variance (ANOVA). When the assumption of sphericity was met, as assessed by Mauchly’s test of sphericity (p > 0.05), the sphericity-assumed results were used. When the assumption of sphericity was violated (Mauchly’s test, p < 0.05), corrections were applied according to the epsilon value: the Greenhouse–Geisser correction was used when epsilon was < 0.75, whereas the Huynh–Feldt correction was applied when epsilon was ≥ 0.75.

Post hoc comparisons between baseline and follow-up measurements were performed using the Bonferroni adjustment for multiple comparisons. A p-value < 0.05 was considered statistically significant.

## Results

A total sample of 104 eyes of 52 patients which met the selection criteria was obtained. During the follow-up period two patients were excluded after undergoing cataract surgery, four patients after changes in their medication or addition of lubricating eye drops, and two patients after losing follow-up. The final sample consisted of 88 eyes of 44 patients, with an average age of 62.9 years and the majority being women (59.1%). The most frequent diagnosis among patients was POAG in 71 eyes (80.7%), most of whom were in the mild stage. (Table 1).

**Table 1:** Sociodemographic variables

	N°	%
Age, years	62.9 ± 8.9	
Sex F M	52 36	59.1 40.9
POAG Preperimetric Mild Moderate Severe	71 14 19 18 20	80.7
		15.9
		21.6
		20.5
		22.7
OHT	17	19.3

POAG, primary open-angle glaucoma; OHT, ocular hypertension; F, female; M, male

When analyzing TCT behavior, a decrease was observed from the baseline average of 537.6 microns to 533.9 microns at 12 months of follow-up, corresponding to a reduction rate of 0.7%, data that was statistically significant. When TCT was broken down by concentric rings, there was a greater decrease in thickness in ring 2 (4.4 microns), followed by rings 3 and 1 at 12 months (3.4 and 3.2 microns, respectively). (Table 2).

**Table 2:** Total corneal thickness (TCT) behavior over one year of follow-up

Group	Days of Follow-up				p-value	Reduction μm
	Baseline	90	180	365		
General (n = 88)						
Ring 1	518.7 ± 31.8	517.4 ± 31.2	516.6 ± 31.4	515.5 ± 30.3^†^	0.001	− 3.2 ± 6.5
Ring 2	534.1 ± 32.2	532.7 ± 31.7	531.2 ± 31.8^†^	529.7 ± 32.6^†^	0.000	− 4.4 ± 11.8
Ring 3	560.0 ± 36.2	558.0 ± 35.8	558.8 ± 33.2	556.6 ± 34.0^†^	0.001	− 3.4 ± 16.0
Ring 4	NC	NC	NC	NC		NC
Mean	537.6 ± 32.9	536.0 ± 32.5	535.6 ± 31.5	533.9 ± 31.5^†^	0.000	− 3.7 ± 8.5
Latanoprost (n = 74)						
Ring 1	522.2 ± 29.5	520.6 ± 29.2	519.8 ± 28.7	518.5 ± 28.3^†^	0.001	− 3.6 ± 6.5
Ring 2	537.9 ± 29.6	536.4 ± 29.4	534.7 ± 29.0^†^	532.9 ± 31.0^†^	0.002	− 5.0 ± 12.5
Ring 3	564.2 ± 34.3	561.9 ± 34.3	562.6 ± 30.3	560.6 ± 31.9	0.291	− 3.6 ± 17.1
Ring 4	NC	NC	NC	NC		NC
Mean	541.4 ± 30.4	539.6 ± 30.4	539.0 ± 28.9	537.3 ± 29.4^†^	0.001	− 4.1 ± 8.8
Brimonidine (n = 8)						
Ring 1	492.6 ± 25.4	492.4 ± 22.9	493.1 ± 24.0	492.1 ± 19.2	0.921	− 0.5 ± 7.9
Ring 2	505.3 ± 27.4	504.1 ± 24.4	505.6 ± 25.7	503.7 ± 21.1	0.898	− 1.5 ± 8.1
Ring 3	529.6 ± 30.5	527.0 ± 26.6	529.1 ± 27.9	526.0 ± 25.3	0.464	− 3.6 ± 7.5
Ring 4	NC	NC	NC	NC		NC
Mean	509.2 ± 27.8	507.8 ± 24.6	509.3 ± 25.8	507.3 ± 21.8	0.713	− 1.9 ± 7.8
Timolol (n = 6)						
Ring 1	510.5 ± 51.7	511.3 ± 51.1	508.0 ± 56.9	508.8 ± 52.1	0.436	− 1.7 ± 3.3
Ring 2	525.4 ± 50.5	525.9 ± 49.6	523.3 ± 55.3	524.9 ± 51.3	0.686	− 0.4 ± 4.0
Ring 3	549.5 ± 50.5	551.1 ± 48.1	552.0 ± 55.5	547.8 ± 50.6	0.742	− 1.7 ± 10.1
Ring 4	NC	NC	NC	NC		NC
Mean	528.5 ± 50.8	529.4 ± 49.5	529.7 ± 53.0	527.2 ± 51.3	0.650	− 1.3 ± 5.6

^*^p-value estimated by repeated-measures ANOVA with sphericity assumed, or adjusted by Greenhouse–Geisser or Huynh–Feldt; ^†^p-value < 0.05 compared with the baseline value, adjusted by Bonferroni

TCT, total corneal thickness; NC, not collected

When evaluating by drug group, TCT behavior at 12 months of follow-up showed a greater decrease in patients treated with latanoprost (4.1 microns), followed by the brimonidine user group (1.9 microns) and finally by the timolol group (1.3 microns). However, only the decrease observed in the latanoprost group reached statistical significance, whereas the changes observed in the brimonidine and timolol groups were not statistically significant.

Regarding the behavior of the TET, the results show an average reduction of 1.7 microns at 12 months of follow-up, results that were statistically significant (p < 0.05). In the evaluation of the epithelium by zone, in all baseline values obtained from each ring, a statistically significant reduction was observed when compared with the measurements at 365 days (p < 0.05). (Table 3).

**Table 3:** Behavior of total epithelial thickness (TET) over one year of follow-up

Group	Days of Follow-up				*p*-value	Reduction μm
	Baseline	90	180	365		
General (n = 88)						
Ring 1	49.2 ± 3.9	48.4 ± 4.3	47.6 ± 4.4^†^	47.1 ± 3.8^†^	0.000	− 2.1 ± 3.5
Ring 2	47.0 ± 3.5	46.0 ± 3.9^†^	45.5 ± 3.8^†^	45.1 ± 3.5^†^	0.000	− 2.0 ± 3.1
Ring 3	43.9 ± 3.3	42.9 ± 3.3^†^	42.8 ± 3.3^†^	42.5 ± 3.0^†^	0.000	− 1.4 ± 3.0
Ring 4	42.2 ± 3.6	41.1 ± 3.1^†^	41.1 ± 3.1^†^	41.0 ± 3.0^†^	0.000	− 1.3 ± 3.4
Mean	45.6 ± 3.2	44.6 ± 3.3^†^	44.2 ± 3.4^†^	43.9 ± 3.0^†^	0.000	− 1.7 ± 2.8
Latanoprost (n = 74)						
Ring 1	49.4 ± 4.0	48.7 ± 4.4	47.9 ± 4.5^†^	47.2 ± 3.9^†^	0.000	− 2.2 ± 3.7
Ring 2	47.1 ± 3.7	46.2 ± 4.0	45.6 ± 4.0^†^	45.2 ± 3.6^†^	0.000	− 1.9 ± 3.2
Ring 3	44.1 ± 3.5	43.0 ± 3.2*	42.9 ± 3.5^†^	42.6 ± 3.1^†^	0.000	− 1.4 ± 3.1
Ring 4	42.4 ± 3.8	41.1 ± 3.2^†^	41.2 ± 3.2	41.2 ± 3.0^†^	0.002	− 1.2 ± 3.6
Mean	45.8 ± 3.3	44.8 ± 3.3^†^	44.4 ± 3.5^†^	44.1 ± 3.1^†^	0.000	− 1.7 ± 3.0
Brimonidine (n = 8)						
Ring 1	48.5 ± 3.4	47.3 ± 4.2	47.4 ± 3.2	47.6 ± 3.7	0.370	− 0.9 ± 2.0
Ring 2	46.8 ± 2.8	45.3 ± 4.1	45.6 ± 2.7	45.1 ± 3.2	0.184	− 1.7 ± 2.1
Ring 3	43.1 ± 2.0	42.1 ± 4.6	43.0 ± 2.3	42.0 ± 2.9	0.666	− 1.1 ± 2.9
Ring 4	41.9 ± 2.3	40.5 ± 3.3	40.9 ± 2.0	40.7 ± 2.6	0.302	− 1.2 ± 1.9
Mean	45.1 ± 2.1	43.8 ± 3.7	44.2 ± 2.3	43.8 ± 2.7	0.352	− 1.2 ± 1.9
Timolol (n = 6)						
Ring 1	47.5 ± 2.9	46.7 ± 3.0	45.2 ± 3.3	44.3 ± 2.2^†^	0.004	− 3.2 ± 1.0
Ring 2	46.0 ± 2.1	44.8 ± 2.1	43.5 ± 2.2	43.3 ± 1.8^†^	0.005	− 2.8 ± 1.3
Ring 3	43.2 ± 1.5	42.6 ± 1.8	41.3 ± 1.2	41.2 ± 2.4	0.030	− 2.0 ± 1.5
Ring 4	40.5 ± 2.0	41.2 ± 2.5	40.1 ± 2.2	38.7 ± 3.1	0.225	− 1.9 ± 1.7
Mean	44.3 ± 1.8	43.8 ± 2.2	42.5 ± 2.2	41.9 ± 1.9^†^	0.021	− 2.4 ± 1.2

^*^p-value estimated by repeated-measures ANOVA with sphericity assumed, or adjusted by Greenhouse–Geisser or Huynh–Feldt; ^†^p-value < 0.05 compared with the baseline value, adjusted by Bonferroni

TET, total epithelial thickness. Reduction: Difference between the 365-day value and the baseline value

A statistically significant average reduction of 1.7 microns in TET was observed in latanoprost users compared to baseline average, with the greatest reduction in rings 1 and 2. In patients receiving brimonidine and timolol, a reduction in all corneal epithelial rings was observed, with the greatest reduction in ring 2 for the brimonidine group and in ring 1 for the timolol group, the latter being statistically significant. Overall, the greatest reduction in TET was achieved in the timolol-treated group, by 2.4 microns compared to baseline numbers, a difference that was also statistically significant. (Table 3).

With regard to the parameters assessed by OSA, the results showed, both overall and in each of the medication groups, a decrease in BUT, tear meniscus height, and lipid layer thickness, as well as an increase in meibomian gland loss at 365 days of follow-up. These changes were statistically significant in the overall analysis and in patients treated with latanoprost. In the case of brimonidine, only statistically significant differences were observed in the increase in meibomian gland loss at 365 days. Patients treated with timolol only showed statistical significance in the decrease in BUT at 180 and 365 days of follow-up. (Table 4).

**Table 4:** Parameters evaluated for the tear film using OSA

Group	Days of Follow-up				p-value	Reduction
	Baseline	90	180	365		
General (n = 88)						
BUT, sec	8.3 ± 1.2	7.9 ± 1.6	7.7 ± 1.4^†^	6.9 ± 0.9^†^	0.000	− 1.4 ± 1.2
Lipid layer, nm	3.8 ± 1.3	3.4 ± 1.3^†^	3.3 ± 1.3^†^	3.0 ± 1.2^†^	0.000	− 1.0 ± 1.6
Meniscus, mm	0.37 ± 0.11	0.35 ± 0.15	0.31 ± 0.11^†^	0.29 ± 0.12^†^	0.000	− 0.08 ± 0.11
GM loss, %	12.0 ± 7.5	12.1 ± 8.9	16.1 ± 10.1^†^	25.2 ± 11.6^†^	0.000	12.6 ± 11.7
Latanoprost (n = 74)						
BUT, sec	8.3 ± 1.1	7.9 ± 1.7	7.6 ± 1.3^†^	6.9 ± 0.8^†^	0.000	− 1.4 ± 1.2
Lipid layer, nm	3.8 ± 1.2	3.5 ± 1.2	3.4 ± 1.3^†^	3.0 ± 1.1^†^	0.000	− 1.1 ± 1.7
Meniscus, mm	0.37 ± 0.10	0.34 ± 0.15	0.31 ± 0.12^†^	0.29 ± 0.12^†^	0.000	− 0.08 ± 0.11
GM loss, %	11.6 ± 7.6	11.2 ± 9.2	15.0 ± 9.7^†^	23.3 ± 10.0^†^	0.000	11.3 ± 10.6
Brimonidina (n = 8)						
BUT, sec	8.0 ± 1.9	8.3 ± 1.0	8.5 ± 2.2	7.0 ± 1.6	0.107	− 1.1 ± 1.2
Lipid layer, nm	4.0 ± 1.4	2.8 ± 1.2	2.5 ± 1.2	3.3 ± 1.5	0.024	− 0.8 ± 1.3
Meniscus, mm	0.35 ± 0.13	0.34 ± 0.13	0.30 ± 0.08	0.30 ± 0.10	0.073	− 0.05 ± 0.07
GM loss, %	13.6 ± 5.7	14.4 ± 5.9	18.9 ± 10.1	35.6 ± 15.2^†^	0.000	22.0 ± 10.2
Timolol (n = 6)						
BUT, sec	8.3 ± 0.3	7.5 ± 0.9	7.3 ± 0.8^†^	7.2 ± 0.6^†^	0.003	− 1.1 ± 0.6
Lipid layer, nm	2.8 ± 1.2	2.3 ± 1.4	2.8 ± 1.2	2.5 ± 1.5	0.518	− 0.3 ± 1.0
Meniscus, mm	0.43 ± 0.10	0.40 ± 0.12	0.33 ± 0.05	0.32 ± 0.08	0.136	− 0.15 ± 0.12
GM loss, %	13.8 ± 9.1	18.2 ± 5.7	22.5 ± 12.5	28.8 ± 15.0	0.098	15.0 ± 20.1

^*^p-value estimated by repeated-measures ANOVA with sphericity assumed, or adjusted by Greenhouse–Geisser or Huynh–Feldt; ^†^p-value < 0.05 compared with the baseline value, adjusted by Bonferroni

OSA, ocular surface analyzer; BUT, tear break-up time; MG, meibomian glands. Reduction: Difference between the 365-day and baseline values

Finally, in the OSDI test assessment, an increase in score was observed at 12 months in all patients compared to baseline numbers. This increase was statistically significant in all controls, both in the overall analysis and in the group treated with latanoprost. Query The group of patients who received timolol obtained the greatest increase in the OSDI test score (8.0 vs. 18.7), followed by patients treated with latanoprost (7.3 vs. 13.6) and brimonidine (7 vs. 8.3), data that also showed statistical significance. (Table 5).

**Table 5:** Evaluation of the OSDI test

Group	Days of Follow-up				*p*-value	Reduction
	Baseline	90	180	365		
General (n = 88)	7.4 ± 6.3	10.6 ± 7.7^†^	11.7 ± 8.8^†^	13.2 ± 11.0^†^	0.000	+ 5.8 ± 9.4
Latanoprost (n = 74)	7.3 ± 6.4	10.7 ± 7.5^†^	11.4 ± 7.7^†^	13.3 ± 10.7^†^	0.000	+ 5.9 ± 9.7
Brimonidina (n = 8)	7.0 ± 5.9	8.0 ± 6.1	10.5 ± 9.9	8.3 ± 8.2	0.177	+ 1.3 ± 4.4
Timolol (n = 6)	8.0 ± 6.3	14.0 ± 13.9	19.0 ± 19.6	18.7 ± 16.0	0.063	+ 10.7 ± 9.8^‡^

^*^p-value estimated by repeated-measures ANOVA with sphericity assumed, or adjusted by Greenhouse–Geisser or Huynh–Feldt; ^†^p-value < 0.05 compared with the baseline value, adjusted by Bonferroni

Reduction: Difference between the OSDI score at 365 days and its baseline value

## Discussion

In the last decade, the advent of new laser technologies, devices, and surgical techniques has reduced the exclusive dependence on hypotensive eye drops for IOP control. The current objective is to maintain stable and long-lasting IOP control, minimizing the need for strict patient adherence to topical treatment [2, 13, 14].

Still, concern remains about the impact of antiglaucoma drugs on the ocular surface and how these may influence treatment adherence. Most previous works describe these alterations in a predominantly subjective manner and have been evaluated in a retrospective manner [4, 5]. This study analyzes the behavior of these medications in the corneal epithelium and the ocular surface in a deeper and more articulated manner, in patients who have never been exposed to hypotensive medications, associating it with a subjective measure that is the patient's perception of dry eye symptoms such as the evaluation of the OSDI test (Table 5).

This study provides for the first time, prospectively, relevant quantitative evidence on the structural changes induced in the cornea once antiglaucoma therapy is initiated. Results demonstrate that, at 12 months, an TCT and TET reduction of 3.7 and 1.7 microns, respectively. (Table 2, 3). This represents a thinning of the TET of approximately 45% of the overall decrease in TCT, suggesting that the effects of topical antiglaucoma medications predominantly affect the corneal epithelium. In contrast, the corneal stroma and endothelium do not appear to be significantly affected, considering that the stroma alone represents 8 to 10 times the thickness of the epithelium [15].

**Fig. 1:**
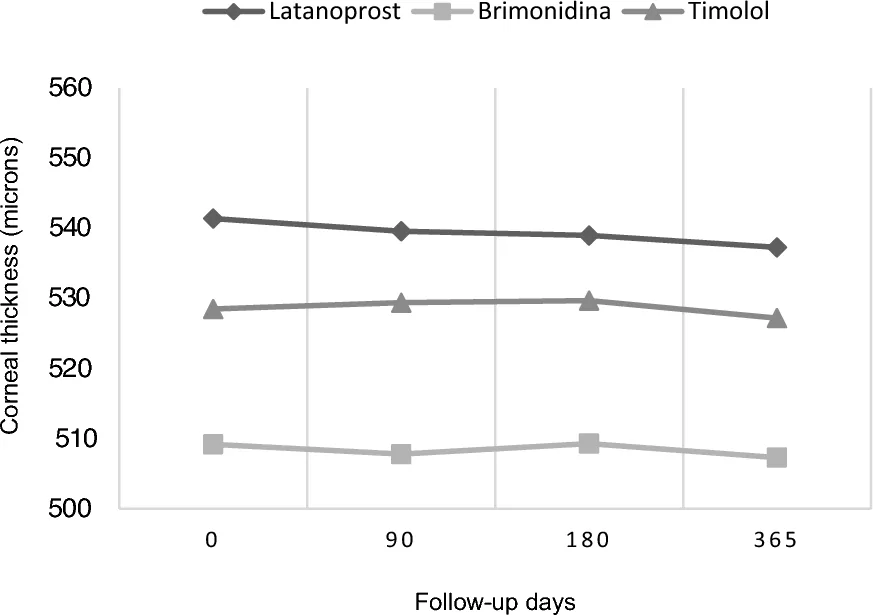
Behavior of total corneal thickness over one year of follow-up

**Fig. 2:**
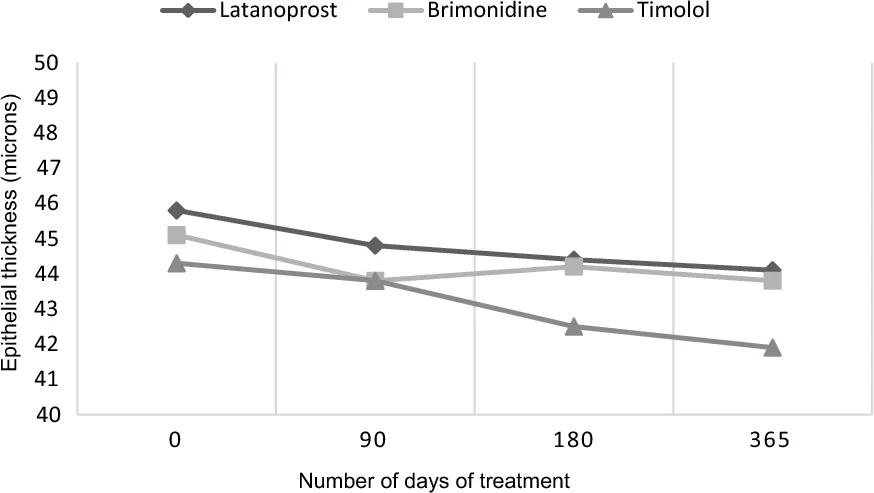
Epithelial thickness behavior after one year of follow-up

The results of the present study demonstrated that the three medications evaluated produce microscopic modifications of the corneal epithelium, inappropriately interfering with the balance that should exist on the ocular surface (Fig. 1, 2). In a general way, a reduction of 1.7 microns in TET compared to baseline was observed, which was statistically significant, suggesting that the toxicity generated by antiglaucoma medications reduces the layers of the corneal epithelium. (Table 3). The results even show a greater loss of these layers in timolol users, followed by latanoprost and finally by brimonidine users. These findings confirm what was reported by Doğan et al. [4], who in a transversal study identified a statistically significant difference in the thickness of the corneal epithelium between users of antiglaucoma medications and healthy subjects (56 vs. 60 microns), which supports the evidence that these treatments negatively affect the integrity of the ocular surface.

In a study performed by Guarnieri et al. [16], the ocular surface was evaluated using the Keratograph 5 M equipment, which uses parameters similar to those evaluated in OSA, showing, in a single measurement, a significant decrease in BUT and tear meniscus height in patients exposed to antiglaucoma medications. In consequence, the present study not only confirms these findings, but broadens the evidence by demonstrating, after 12 months of follow-up, a statistically significant reduction in BUT and tear meniscus height, as well as a loss of the lipid layer, all associated with a progressive loss of the Meibomian glands, resulting in poor quality and poor stability of the tear film, favoring its evaporation (Table 4).

The most accepted hypothesis indicates that hyperkeratinization of the terminal ducts is the main mechanism by which obstructive meibomian gland dysfunction occurs, which is generally associated with aging, hormonal imbalance and drug toxicity [17, 18]. Zhang et al. [19] showed significant loss of epithelial cells in the meibomian gland ducts after prolonged exposure to timolol, specially when its use exceeded 12 months. On their part, Uzunosmanoglu et al. [20], in a comparative study, reported a greater loss of glandular ducts in patients treated with prostaglandin analogues, in comparison to those using other antiglaucoma medications, a difference that was statistically significant after one year of treatment. In line with these findings, the results of the present study confirm a progressive decline in the functionality of the Meibomian glands throughout follow-up, observing an average loss of 12.5%, which reached 25.5% at the end of the observation period, a statistically significant fact. Nevertheless, unlike previous studies, in our sample the greatest loss was observed in the groups treated with timolol and brimonidine, while patients receiving latanoprost showed less impairment. This discrepancy may be explained by the small size of the timolol subgroup, the non-randomized treatment allocation, and the presence of BAK in all formulations, factors that preclude attributing the observed effect solely to the active ingredient. Furthermore, beta-blockers may reduce tear secretion, a mechanism that could contribute to the observed ocular surface deterioration and warrants confirmation in studies with larger sample sizes and preservative-free formulations.

Several studies have documented higher scores on the OSDI test in patients using antiglaucoma drugs when compared with those who do not use them [7, 10, 21]. In our study, prior to the start of treatment, the OSDI score was within normal limits; at the end of follow-up, an increase in the score was observed in all patients, a statistically significant fact. Our results suggest that patients treated with timolol not only reported the highest OSDI scores, but also a greater decrease in TET, which suggests that epithelial damage is directly proportional to the symptoms reported by patients. On the other hand, the lower score observed in the group treated with brimonidine could be explained by its vasoconstrictor effect, inducing a subjective sensation of greater ocular comfort with less hyperemia, therefore attenuating the symptoms perceived by the patient [22]. Furthermore, it should be considered that as epithelial thinning progresses, greater exposure of the nerve endings extending into the superficial epithelial layers may occur, which could contribute to worsening ocular surface symptoms [23].

Finally, the present study—which, as far as we know, is the first with a prospective approach—evaluated the effect of three antiglaucoma drugs containing benzalkonium chloride as a preservative on the ocular surface. Although this relationship has been previously studied, our work is the first to analyze how changes in the ocular surface evolve over time once antiglaucoma treatment is established. Nevertheless, we consider that future studies will require direct comparisons of medications with and without preservatives, in order to more accurately estimate potential damage to the ocular surface, optimizing symptomatic management in patients with glaucoma and improving pharmacological adherence.

## Conclusion

This study provides prospective evidence that the use of first-line antiglaucoma medications containing preservatives is associated with corneal epithelial alterations, as demonstrated by quantitative analysis using Cirrus 6000 OCT and OSA. These changes were accompanied by a significant deterioration in tear film stability, including alterations in the lipid layer, tear breakup time (BUT), tear meniscus height, and meibomian gland density, thereby compromising ocular surface integrity. Furthermore, a direct association appears to exist between epithelial thinning and the increase in ocular discomfort symptoms reported by patients through the Ocular Surface Disease Index (OSDI), which may negatively affect both quality of life and treatment adherence.

## Data Availability

No datasets were generated or analysed during the current study.
